# Characterisation of *Salmonella* Enteritidis ST11 and ST1925 Associated with Human Intestinal and Extra-Intestinal Infections in Singapore

**DOI:** 10.3390/ijerph19095671

**Published:** 2022-05-06

**Authors:** Kyaw Thu Aung, Wei Ching Khor, Kar Hui Ong, Wei Ling Tan, Zhi Ning Wong, Jia Quan Oh, Wai Kwan Wong, Brian Zi Yan Tan, Matthias Maiwald, Nancy Wen Sim Tee, Timothy Barkham, Tse Hsien Koh, Anders Dalsgaard, Swaine L. Chen, Joergen Schlundt, Lee Ching Ng

**Affiliations:** 1 National Centre for Food Science, Singapore Food Agency, Singapore 718837, Singapore; khor_wei_ching@sfa.gov.sg (W.C.K.); ong_kar_hui@sfa.gov.sg (K.H.O.); tan_wei_ling@sfa.gov.sg (W.L.T.); wong_zhi_ning@sfa.gov.sg (Z.N.W.); andrew_oh@sfa.gov.sg (J.Q.O.); 2 Environmental Health Institute, National Environment Agency, Singapore 138667, Singapore; ng_lee_ching@nea.gov.sg; 3 Nanyang Technological University Food Technology Centre (NAFTEC), Singapore 637459, Singapore; adal@sund.ku.dk (A.D.); joergenschlundt@gmail.com (J.S.); 4 School of Chemical and Biomedical Engineering, Nanyang Technological University, Singapore 637459, Singapore; 5 School of Biological Sciences, Nanyang Technological University, Singapore 637551, Singapore; 6 Animal & Veterinary Service, National Parks Board, Singapore 718827, Singapore; wong_wai_kwan@nparks.gov.sg (W.K.W.); briantzyan@gmail.com (B.Z.Y.T.); 7 Department of Pathology and Laboratory Medicine, KK Women’s and Children’s Hospital, Singapore 229899, Singapore; matthias.maiwald@singhealth.com.sg; 8 Department of Microbiology and Immunology, Yong Loo Lin School of Medicine, National University of Singapore, Singapore 117545, Singapore; 9 Duke-NUS Graduate Medical School, National University of Singapore, Singapore 169857, Singapore; 10 Department of Laboratory Medicine, National University Hospital, Singapore 119074, Singapore; nancy_tee@nuhs.edu.sg; 11 Department of Laboratory Medicine, Tan Tock Seng Hospital, Singapore 308433, Singapore; timothy_barkham@ttsh.com.sg; 12 Department of Pathology, Singapore General Hospital, Singapore 169856, Singapore; koh.tse.hsien@singhealth.com.sg; 13 Department of Veterinary and Animal Sciences, University of Copenhagen, 1871 Frederiksberg, Denmark; 14 NUHS Infectious Diseases Translational Research Programme and Department of Medicine, Division of Infectious Diseases, Yong Loo Lin School of Medicine, National University of Singapore, 1E Kent Ridge Road, NUHS Tower Block, Singapore 119228, Singapore; slchen@gis.a-star.edu.sg; 15 Laboratory of Bacterial Genomics, Genome Institute of Singapore, 60 Biopolis Street, Singapore 138672, Singapore

**Keywords:** *Salmonella* Enteritidis, sequence type, salmonellosis, epidemiology, antimicrobial resistance, human, food, farm environment, integrated surveillance

## Abstract

*Salmonella* Enteritidis is a major foodborne pathogen worldwide. In this study, a total of 276 *S. enteritidis* isolates, collected between 2016 and 2017 from human, food and farm/slaughterhouse samples, were studied to enhance the understanding of the epidemiology of human salmonellosis in Singapore. Results showed all 276 isolates belonged either to ST1925 (70.3%) or ST11 (29.7%), with ST11 being significantly more frequent in extra-intestinal isolates and chicken isolates. Food isolates, most of which were from poultry, showed the highest prevalence of resistance (33–37%) against beta-lactams or beta-lactams/beta-lactamase inhibitor combination (ampicillin, piperacillin and ampicillin/sulbactam). The analysis showed the detection of genes associated with resistance to aminoglycoside genes (99.6%), tetracycline (55.1%), and beta-lactams (14.9%) of all isolates. Nine types of plasmids were found in 266 isolates; the most common incompatibility group profiles were IncFIB(S)-IncFII(S)-IncX1 (72.2%) and IncFIB(S)-IncFII(S) (15.8%). Most plasmid harbouring isolates from chicken (63.6%, 14/22) and from human (73.8%, 175/237) shared the same plasmid profile (IncFIB(S)-IncFII(S)-IncX1). SNP analysis showed clustering of several isolates from poultry food products and human isolates, suggesting phylogenetic relatedness among these isolates. Lastly, this study provides important epidemiological insights on the application of phenotypic and next-generation sequencing (NGS) tools for improved food safety and public health surveillance and outbreak investigation of *S.* *enteritidis*.

## 1. Introduction

*Salmonella* Enteritidis is one of the most prevalent serovars causing foodborne human salmonellosis worldwide, frequently associated with the consumption of poultry and eggs [1,2,3]. Similar to other non-typhoidal serovars, *S.* Enteritidis typically causes gastrointestinal-related symptoms, although invasive strains of *Salmonella* have emerged as a prominent cause of bloodstream and other invasive infections worldwide [4,5,6]. In addition to its ability to cause infections, *S*. Enteritidis has been increasingly reported to be resistant to commonly used antimicrobials, limiting therapeutic choices for treating severe infections [3,7,8]. Hence, the World Health Organization (WHO) has listed *Salmonella* in the global priority pathogen list of antibiotic-resistant bacteria [9].

*Salmonella*, a Gram-negative, facultative anaerobic bacterium, belongs to the family of *Enterobacteriaceae* [10]. *Salmonella* can live in the intestinal tract of animals, including food animals, and can be widely distributed in natural and man-made environments [11,12,13,14]. Antimicrobial-resistant *Salmonella* is known as a major public health challenge arising from mis- or over-use of antibiotics in human and animal sectors, and can be transmitted to humans throughout the food chain [15]. Antimicrobial-resistant genes found in *Salmonella* can further enhance their ability of biofilm formation for persistent contamination and survival in the environment [16]. *Salmonella* can typically be acquired through the consumption of contaminated foods including poultry, and eggs [17]. The risk of contamination and subsequent infections can be increased by improper cooking and handling practices as well as by cross-contamination and time-temperature abuse of ready-to-eat food [18,19].

In Singapore, non-typhoidal salmonellosis has been a notifiable foodborne disease since 2008. In 2016, the reported incidence rate of non-typhoidal salmonellosis (39.4 per 100,000 population) was approximately three times higher than that reported in 2008 (14.8 per 100,000 population) [20]. Specifically, *S*. Enteritidis has consistently been a frequent serovar associated with >20% of human cases in Singapore [21]. Between 2012 and 2016, there were 1152 reported *S.* Enteritidis human infections, which would represent a considerable burden of diseases, in Singapore [18]. In addition, previous studies reported that *S.* Enteritidis was one of the predominant serovars in raw poultry products and eggs products as well as in cooked or ready-to-eat dishes containing poultry or eggs as ingredients [21,22]. The same studies also reported phenotypic antimicrobial resistance profiles in *S.* Enteritidis [22]. Another study investigated the use of next-generation sequencing approach to elucidate genetic relatedness of epidemiological linked *S.* Enteritidis isolates from suspected outbreaks [23]. These prior data informed the epidemiological significance of *S.* Enteritidis in Singapore. Notwithstanding, to our knowledge, phenotypic and genotypic characterisation of *S.* Enteritidis isolates available from humans and the food chain are limited.

To gain a deeper understanding of the epidemiology of human salmonellosis in Singapore, this study sequenced the genomes of 276 *S.* Enteritidis isolates from human, food and farm/slaughterhouse samples in 2016–2017. We identified sequence types, virulence factors, and phylogenetic relationships. In addition, the study examined and compared antimicrobial resistance genotypes with the phenotypes determined by broth micro-dilution.

## 2. Materials and Methods

### 2.1. Collection of Salmonella Isolates

A total of 276 *S.* Enteritidis isolates (human, *n* = 246; food, *n* = 27; farm and slaughter house environment, *n* = 3), isolated between 2016 and 2017 (study period), were included in this study. The human isolates, contributed by three public hospitals, namely KK Women’s and Children’s Hospital, Singapore General Hospital and Tan Tock Seng Hospital, were retrieved from bacterial biobanks, which comprise isolates from specimens from inpatients or notified foodborne salmonellosis cases, whereas the food and farm/slaughterhouse isolates available during the same study period were retrieved from the archived bacterial biobanks of the national food safety, animal and environmental health laboratories. The details of isolates are shown in Table 1. Isolates were streaked on tryptone soya agar (Oxoid, UK) to confirm purity and for further characterisation as described below.

### 2.2. Genomic DNA Extraction for Whole Genome Sequencing (WGS) and Analysis

Whole-genome sequencing was performed by the Genome Institute of Singapore and the Agency for Science, Technology and Research. Briefly, 1 µL loopful of bacterial culture from glycerol vial was inoculated into the 5 mL of Universal Pre-enrichment Broth (Acumedia, Lansing, MI, USA) for overnight (18–24 h) incubation at 37 °C. After the incubation, 1 mL (approximately containing >10^5^ cells) of each *S*. Enteritidis overnight culture in Universal Pre-Enrichment Broth broth was centrifuged. Next, the bacterial cells were lysed by using enzymatic lysis buffer at 37 °C for 45 min, followed by extraction using the DNeasy Blood and Tissue Kit (QIAGEN, Valencia, CA, USA) according to the manufacturer’s instructions. Genomic DNA shearing was performed by using an M220 Focused Ultrasonicator (Covaris, Woburn, MA, USA), and library preparation was performed by using NEBNext^®^ Ultra™ DNA Library Prep Kit (NEB, Ipswich, MA, USA). Samples were then sequenced by using a HiSeq 4000 sequencer (Illumina, San Diego, CA, USA) with 2 × 151-bp reads.

All primary sequence analysis was performed by the Genome Institute of Singapore Efficient Rapid Microbial Sequencing (GERMS). Reference-based analyses were performed by using strain LT2 (Genbank accession NC_003197.2). FASTQ files were mapped by using Burrows-Wheeler Aligner version 0.7.17 software [24]. Indel realignment and single-nucleotide polymorphism (SNP) calling was performed by using Lofreq* version 2.1.2 with default parameters [25]. Approximately Maximum-Likelihood trees were made using FastTree 2.1.10 [26]. All phylogenetic trees were visualised with GGTREE version 3.2 in R version 3.2.2 (https://www.R-project.org) [27]. Multilocus sequence type (MLST) predictions were made by using SRST2 version 0.1.8 for Illumina sequenced strains or manually by using BLASTN for fully assembled reference sequences, using the MLST database (http://pubmlst.org/salmonella (accessed on 1 January 2021)) [28,29,30]. Resistance gene prediction was performed using the ARGannot resistance gene database included with SRST2 [31]. Serotype prediction was performed using the SeqSero programme [32]. Virulence gene prediction was made using VFDB as a database as recommended by SRST2 [30]. Additionally, the assembled genome sequences were analysed for the identification of Salmonella Pathogenicity Islands (SPIs) and plasmids using Centre for Genomic Epidemiology (CGE)’s SPIFinder 1.0 (https://cge.cbs.dtu.dk/services/SPIFinder/ (accessed on 1 January 2021)) and PlasmidFinder 2.1 (https://cge.cbs.dtu.dk/services/PlasmidFinder/ (accessed on 1 January 2021)), respectively. Raw sequence data was deposited into Genbank under Bioproject accession number PRJNA810928.

### 2.3. Phenotypic Antimicrobial Susceptibility Testing

Antimicrobial susceptibility testing (AST) was performed by the broth microdilution method to determine the minimum inhibitory concentration (MIC). MicroScan Neg MIC Panel Type 44 (Beckman Coulter, Inc., Brea, CA, USA) was used following the manufacturer’s instructions. The antimicrobial susceptibility profiles of each isolate were determined in accordance with the available MIC interpretations published by the Clinical and Laboratory Standards Institute (CLSI) or EUCAST as appropriate. The isolates with MICs in the sensitive and intermediate range were categorised as susceptible to avoid overestimation of resistance. Isolates that were resistant to more than or equal to three antimicrobial classes were classified as multi-drug-resistant strains.

All isolates were subjected to antimicrobial susceptibility testing against 28 antimicrobials belonging to nine antimicrobial classes: amikacin; amoxicillin/k clavulanate; ampicillin; ampicillin/sulbactam; aztreonam; cefepime; cefotaxime; cefoxitin; ceftazidime; cefuroxime; chloramphenicol; ciprofloxacin; colistin; doripenem; ertapenem; fosfomycin; gentamicin; imipenem; levofloxacin; meropenem; minocycline; nitrofurantoin; norfloxacin; piperacillin; piperacillin/tazobactam; tetracycline; tobramycin; trimethoprim/sulfamethoxazole. Extended spectrum beta lactamase (ESBL) enzyme production was confirmed using a combination of cefotaxime/k clavulanate and ceftazidime/k clavulanate.

### 2.4. Statistical Calculation and Analysis

Statistical calculations were done using the SPSS (IBM SPSS Statistics for Windows, Version 27.0; IBM Corp., Armonk, NY, USA). Associations between virulence gene markers, antimicrobial resistance, sequence types and potential invasiveness (observation in extra-intestinal isolates) of the human isolates were calculated using Pearson’s Chi-squared test or Fisher’s exact test. Z-scores for two population proportions were calculated using https://www.socscistatistics.com/tests/ztest/default2.aspx (accessed on 1 January 2021).

## 3. Results

All *S.* Enteritidis isolates in this study belonged to ST1925 (70.3%, *n* = 194) and ST11 (29.7%, *n* = 82) (Table 1). Of the human isolates, 69.5% (171/246) and 30.5% (75/246) were from intestinal (stool) and extra-intestinal (blood and other organs) samples, respectively, and were ST1925 (*n* = 184) or ST11 (*n* = 62) (Table 1). The proportion of ST11 (41.9%, 26/62) associated with the potentially invasive isolates from extra-intestinal samples was significantly higher than the proportion of ST1925 (26.6%, 49/184) (*p* < 0.05 by Z-score). Statistical analysis showed a significant association between ST11 and the isolates being from the extra-intestinal samples (Chi Square 5.1257, *p* < 0.05).

Of the 27 food isolates, 23 (85.2%) were from raw chicken meats (chilled, frozen and minced) and the rest were from raw duck meats (*n* = 2) and cooked food (*n* = 2). A total of 17 of the 23 (73.9%) chicken isolates belonged to ST11. Of the three isolates from farm and slaughterhouse environments, two isolates belonged to ST1925 and one isolate from a water sample belonged to ST11 (Table 1).

### 3.1. Phenotypic Antimicrobial Resistance (Broth Micro Dilution)

All *S.* Enteritidis isolates in this study were susceptible to amikacin, piperacillin/tazobactam, imipenem, meropenem, doripenem, ertapenem, ciprofloxacin, levofloxacin, trimethoprim/sulfamethoxazole, chloramphenicol and colistin. Almost all isolates, (98.9%; *n* = 273) were resistant to at least one antimicrobial tested. One human intestinal and three chicken meat isolates were susceptible to all antimicrobials tested. The isolates were most commonly resistant to tetracyclines (tetracycline 54.7%; minocycline 35.5%), penicillins (ampicillin, 16.6%; piperacillin, 15.2%), fluoroquinolones (norfloxacin 15.9%) and beta lactam/beta lactamase inhibitor combination (ampicillin/sulbactam 14.8%). Two (0.7%) ESBL-positive isolates were detected from one intestinal and one extra-intestinal (knee) sample; both isolates were multidrug resistant.

**Figure 1:**
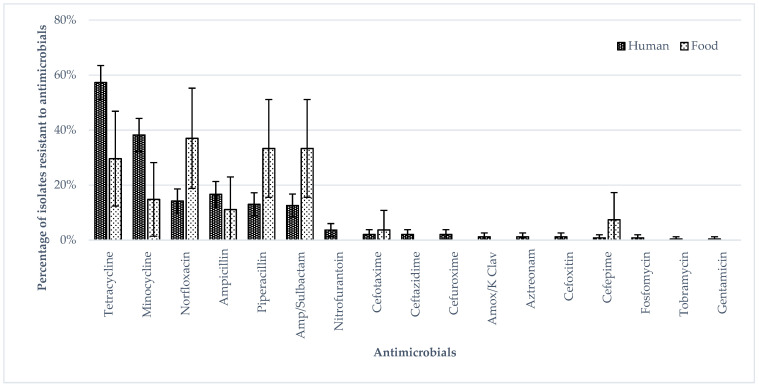
Phenotypic antimicrobial resistance in *S.* Enteritidis isolates, comparing human and food isolates.

**Figure 2:**
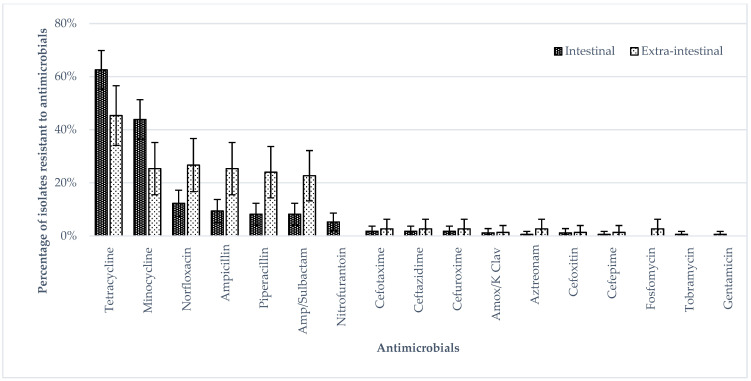
Phenotypic antimicrobial resistance in *S.* Enteritidis, comparing intestinal and extra-intestinal human isolates.

**Figure 3:**
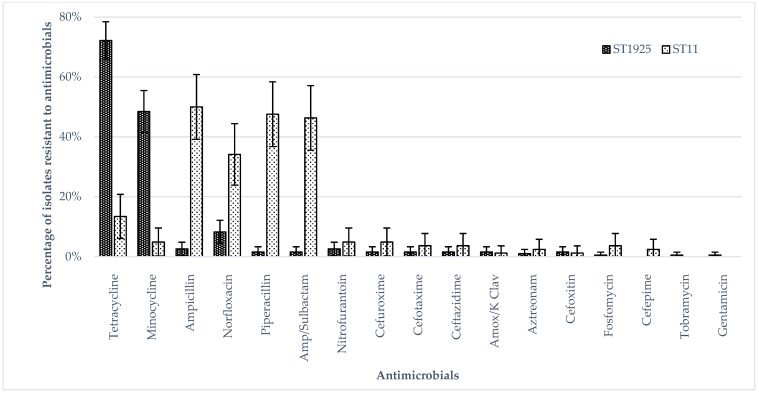
Phenotypic antimicrobial resistance in ST1925 and ST11 isolates.

Figure 1 shows the antimicrobial susceptibility of the human and food isolates with the highest percentages of isolates resistant to minocycline, norfloxacin, tetracycline, piperacillin, ampicillin and ampicillin/sulbactam. The percentages of human isolates resistant to tetracycline and minocycline were higher than those of food isolates. The percentages of extra-intestinal isolates resistant to norfloxacin, ampicillin, piperacillin and ampicillin/sulbactam were higher than those of intestinal isolates, whereas the percentages of intestinal isolates resistant to tetracycline and minocycline were higher than those of extra-intestinal isolates (*p* < 0.05 by Fisher’s Exact test) (Figure 2). The percentages of ST11 isolates resistant to ampicillin, norfloxacin, piperacillin and ampicillin/sulbactam were higher than those of ST1925 isolates (Figure 3) (*p* < 0.05 by Fisher’s Exact test) whereas ST1925 isolates had higher resistance towards tetracycline and minocycline.

There were 56 multi-drug-resistant (MDR) isolates: 44/246 (17.9%) from human, 11/27 (40.7%) from food and 1/3 (33.3%) from a farm/slaughterhouse environment (environmental swab). Twenty-one (21/75, 28.0%) and 23 (23/171, 13.5%) isolates from extra-intestinal and intestinal isolates were found to be MDR. There was a significant association between MDR and the isolates being from the extra-intestinal samples (Chi Square 7.633, *p* < 0.05).

### 3.2. Genotypic Antimicrobial Resistance, Virulence, SPI and Plasmid Profiles

Genomic analysis showed that 99.6% of the isolates carried at least 1 antimicrobial resistance gene. The highest number of antimicrobial resistance genes detected in an MDR intestinal isolate from a human stool sample was five.

Aminoglycoside resistance genes were the most detected in 99.6% (*n* = 275) of the isolates, followed by tetracycline resistance genes (152, 55.1%), beta-lactam resistance genes (41, 14.9%), folate pathway inhibitor resistance genes (2, 0.7%), fluoroquinolones resistance genes (1, 0.4%) and polymyxin resistance genes (1, 0.4%) (Table 2). A colistin resistance gene (*mcr1*) was detected in an ST1925 isolate from a human stool sample which was, however, phenotypically susceptible (>4 µg/mL) to colistin in MIC susceptibility testing [33,34]. Similarly, a fluoroquinolone resistance gene was detected in a ST11 isolate from a human stool sample but the isolate was phenotypically susceptible to fluroquinolones. One ST11 isolated from a water sample from farm/slaughterhouse environment, and one ST1925 from a human stool were detected that had folate inhibitor pathway resistance genes but showed phenotypic susceptibility to folate pathway inhibitors tested in the study.

Statistical analysis showed a significant positive association between the detection of *TEM1D_bla* gene and the isolates being from extra-intestinal samples (Chi Square 10.710 and 5.239, *p* < 0.05). Conversely, a significant negative association was observed between the detection of *tetA* gene and the isolates being from the extra-intestinal samples (Chi Square 8.020, *p* < 0.05).

Point mutations in the quinolone resistance-determining regions (QRDRs) of *gyrA*, *gyrB*, *parC*, and *parE* were observed among *S*. Enteritidis isolates (33.7%, 93/276). The most frequent mutation in *gyrA* is D87Y occurring in 47.3% of the isolates (44/93), followed by D87G (16.1%, 15/93), S83Y (14.0%, 13/93), S83F (8.6%, 8/93) and D87N (4.3%, 4/93). All isolates harbouring mutations in *gyrA* were found to be resistant or having reduced susceptibility against norfloxacin (fluoroquinolone), suggesting the likely association of *gyrA*-associated mutations in response to antimicrobial selection pressure [35]. Mutations in *parE* (4/93, A61T), *pmrB* (3/93, H164Y), *gyrB* (1/93, T717N), and *parC* (1/93, T57S) were less frequent among *S.* Enteritidis in this study.

All isolates, except one from a chilled minced chicken sample, were detected with virulence genes with an average number of 58.9 virulence genes. There were significant positive associations between the detection of *X1* or *X1TaxC* genes and the isolates being from the human extra-intestinal (invasive) samples (Chi Square 5.602 and 5.239, *p* < 0.05).

All *S.* Enteritidis isolates contained *Salmonella* pathogenicity island, SPI-1, SPI-3 to SPI-5, SPI-13, SPI-14 and centisome 63 pathogenicity island, C63PI-1; with the exception of five isolates which contained the following SPIs, respectively (one human-intestinal isolate with SPI-3, SPI-8, SPI-13, SPI-14; one human extra-intestinal isolate, one chicken isolate, and one farm and slaughter house environment isolate with SPI-1, SPI-3, SPI-5, SPI-13, SPI-14 and C63PI; one human-intestinal isolate with SPI-3, SPI-8, SPI-13, and SPI-14; and one chicken isolate with SPI-1, SPI-3 to SPI-5, SPI-12 to SPI-14 and C63PI). There were no major differences in SPI profiles between the intestinal and extra-intestinal isolates.

Of 276 *S.* Enteritidis isolates, 266 isolates (237 from human; 22 from chicken; 3 from a farm/slaughterhouse environment; 2 from duck; and 2 from cooked or RTE food) carried plasmids. The most common plasmid profiles were IncFIB(S)-IncFII(S)-IncX1 (72.2%, 192/266), IncFIB(S)-IncFII(S) (15.8%, 42/266), and ColpVC-IncFIB(S)-IncFII(S)-IncX1 (6.0%, 16/266). These plasmids can encode virulence and antimicrobial resistance genes, and can contribute to the bacterial diversification and adaptation through horizon gene transfer [36,37,38]. In addition, plasmids can serve as epidemiological markers of various bacterial strains that are useful for surveillance and outbreak investigation of potential emergence of virulent subtypes [39,40]. Among 22 *S.* Enteritidis isolates from chicken, the majority (63.6%, 14/22) shared a matching plasmid profile (IncFIB(S)-IncFII(S)-IncX1) with the human isolates (73.8%, 175/237), suggesting their limited host reservoirs [41,42].

### 3.3. Phylogenetic Analysis

**Figure 4:**
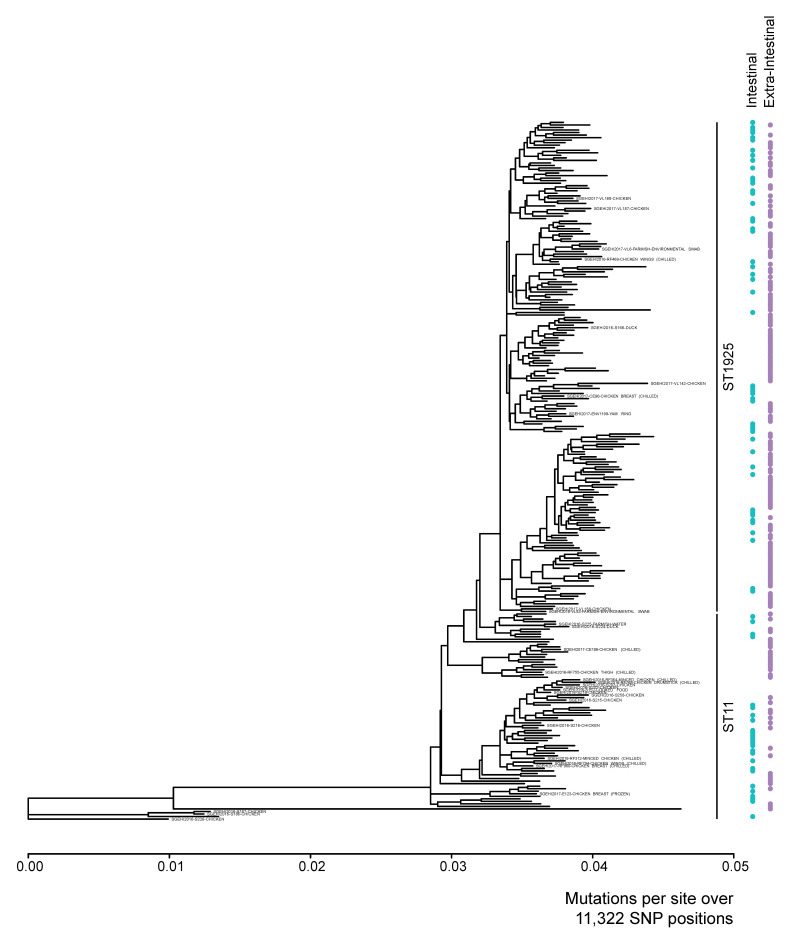
Maximum-likelihood SNP trees of 276 *S*. Enteritidis isolates. Food, and farm/slaughter house environment isolates were labelled on the tree, whereas non-labelled isolates were human isolates.

Figure 4 shows the phylogenetic relatedness of 276 *S*. Enteritidis isolates based on whole-genome SNP analysis. Overall, WGS further separated the *S*. Enteritidis isolates that belonged to the same MLST sequence type with no overlap between the two sequence types. No distinct phylogenetic clusters of isolates between human extra-intestinal (pink circles) and intestinal isolates were observed. Two isolates from chicken meat (SGEHI2017-VL187, and SGEHI2017-VL168) and one isolate from duck meat (SGEHI2016-S166) were clustered with the human isolates (less than 150 SNPs difference). One isolate from duck meat (SGEHI2016-S224) clustered (81 SNPs difference) with the isolate from a swab from a farm/slaughter house (SGEHI2016-S225). Several chicken meat isolates (*n* = 10) were clustered (1 to 159 SNPs difference) with human isolates.

## 4. Discussion

In this study, we investigated 276 *S*. Enteritidis isolates from various sources. A relatively smaller proportion of isolates from food and farm/slaughterhouse samples from 2016–2017 were available compared to isolates from human samples. This was due to an overall downward trend in the relative occurrence of *S*. Enteritidis among *Salmonella* isolates from different food categories, where *S.* Enteritidis was more frequently isolated from fresh than frozen chicken meat samples, as reported by a previous study from Singapore [18].

### 4.1. Sequence Type in S. Enteritidis Isolates

Our finding of *S*. Enteritidis as the most common serovar associated with nontyphoidal salmonellosis is in line with the global trend reported by the US Centers for Diseases Control and Prevention and European Food Safety Authority [43,44]. In this study, only two sequence types, ST11 and ST1925, were identified among *S*. Enteritidis isolates from human, food and farm/slaughterhouse samples. In contrast, studies showed, while ST11 and ST1925 were the predominant sequence types, there had been other sequence types less frequently found among *S.* Enteritidis isolates in food, animals and humans in various countries [45,46,47,48,49,50,51].

Both sequence types ST11 and ST1925 were known to be geographically widespread and had been reported in various sectors (human, food, chicken slaughterhouses) [48,52,53]. The high prevalence of ST11 among cases has been reported, with 95% of *S.* Enteritidis isolated in England and Wales between April 2014 and March 2015 belonging to ST11. ST11 accounted for 89% of the 17,867 *S.* Enteritidis entries in the EnteroBase database, whereas ST1925, the single locus variant of ST11, had 84 entries in the EnteroBase (http://enterobase.warwick.ac.uk (accessed on 1 January 2021)). In contrast, findings from this study showed that 70% of the *S.* Enteritidis isolates belonged to ST1925. A similar trend was reported in an earlier study in Singapore where all *S.* Enteritidis isolates from suspected outbreaks belonged to ST1925 [23]. In addition, ST1925 strains were previously reported in various types of cooked or ready-to-eat food samples collected from the retail food establishments in Singapore [52]. These suggest the epidemiological significance of ST1925, and possible roles of retail food contamination in the transmission dynamics of ST1925 in the local settings.

However, although ST11 forms a minority of the isolates among cases in Singapore, it was more likely to be found among human extra-intestinal isolates, when compared with ST1925. The finding was consistent with previous studies that showed ST11 as one of the sequence types most commonly associated with invasive salmonellosis in Asia and Africa [51,54]. Studies showed that ST11 was a predominant sequence type found in avian, chicken and chicken products in Malaysia [45,46,50]. Another study showed ST11 infections reportedly linked to eggs and egg products related to a multi-country outbreak in five European Union/European Economic Area countries and the United Kingdom in 2021 [55]. The occurrence of the potentially invasive sequence type in the chicken products (74% of the chicken isolates), coupled with the emergence of antimicrobial resistance could be of public health concern and should be closely monitored. The finding emphasises the importance of reducing the *Salmonella* prevalence in these chicken products before they reach the retail shelves, as well as proper handling and thorough cooking of chicken meat to reduce the food safety risk of *Salmonella* and other foodborne pathogens.

### 4.2. Antimicrobial Resistance Phenotype in S. Enteritidis Isolates

Although *Salmonella* usually causes self-limiting gastroenteritis without the need of antimicrobials, it may lead to severe infection in immunocompromised patients who require antimicrobial therapy [4]. Our finding of human *S.* *enteritidis* isolates showing the highest resistance rate against tetracycline may be of concern as tetracycline has an extensive application in human and veterinary medicine as well as in agriculture and aquaculture sectors [56]. Wang et al., (2019) reported a surge in tetracycline-resistance in *Salmonella* isolates from humans which coincided with a similar surge in food-animal isolates, suggesting the roles of food animals in serving as reservoirs of tetracycline-resistant organisms to humans [57]. Tetracyclines are being widely used as growth promoters in animal husbandries and are identified by the WHO as highly important for human health, thus monitoring of the efficacy of these antibiotics is paramount [9,58].

None of the *S.* Enteritidis isolates in this study exhibited resistance to colistin. In addition, resistance of *Salmonella* isolates in this study against those WHO’s critically important antimicrobials were at relatively low levels (<5%) suggesting that, for now, effective antimicrobials for the treatment of salmonellosis remain available. In contrast, the higher percentages of isolates resistant to ampicillin, norfloxacin, piperacillin and ampicillin/sulbactam of 15 to 20%, especially among invasive isolates from extra-intestinal samples, call for close monitoring and action to reverse the trend [59,60,61].

Food isolates, mainly from poultry products, showed the greatest resistance against beta-lactams or beta-lactams/beta-lactamase inhibitor combination (ampicillin, piperacillin and ampicillin/sulbactam) at 33–37%. The similar resistance patterns in poultry were reported in countries where Singapore imports most of its poultry products from [62]. *S*. Enteritidis isolated from raw, frozen and stuffed chicken associated with multistate disease outbreaks in the United States were resistant to ampicillin [63]. In Malaysia, a relatively high percentage (72.7%) of *S*. Enteritidis isolated from raw chicken meats from wet markets and supermarkets showed ampicillin resistance, although all showed inhibited growth in the presence of beta-lactamase inhibitor [64].

This study further observed statistically significant associations between multidrug resistance, sequence type (ST11) and the isolates from extra-intestinal samples. These suggest that antimicrobial resistance in *Salmonella* may associate with an increased risk of invasiveness and more severe infections. While such hypothesis warrants further investigations, findings from this study highlight the importance of integrated analysis of both antimicrobial resistance and virulence factors, in order to enhance our understanding of the impact of such evolution on public health.

### 4.3. Phylogenetic Relation between Some Human, Food and Farm/Slaughterhouse Isolates

Whole-genome sequence (WGS) analysis offers a greater resolution for differentiating bacterial isolates than other microbial subtyping methods such as serotyping and MLST. In agreement with other work, this study found that WGS-based SNP analysis was able to further discriminate *S*. Enteritidis isolates belonging to the same sequence type, separating phylogenetic unrelated isolates from related ones [23,65,66].

The clustering of human isolates with food isolates from poultry food products and one isolate from an environmental swab from a farm/slaughterhouse show a close phylogenetic relationship between the isolates, suggesting the possibility of common chains of transmission. This finding, together with previous evidence, indicates that poultry meat likely plays an important role in the epidemiology of *S*. Enteritidis in Singapore [18]. More specific epidemiological information, with a higher number of the isolates, is required to better understand the disease transmission pathway. The use of whole genome sequencing on a larger collection of *Salmonella* isolates can be further complemented by the application of mathematical models, coupling with the machine-learning algorithms capable of recognizing patterns in complex datasets, for the better estimation of the proportion of human salmonellosis attributable to various food, animals and environmental sources.

## 5. Conclusions

Despite control efforts, *S*. Enteritidis remains a significant cause of foodborne illnesses worldwide. To this end, the study integrated microbial and molecular subtyping insights generated from cross-sectoral ‘One Health’ efforts to offer a step forward in understanding the epidemiology of human salmonellosis. Our results highlight the phylogenetic link between the isolates from human and poultry, and the importance of monitoring the emergence of antimicrobial resistance, especially among ST11 isolates, which are known to be more likely to be invasive. The study is limited by the number of isolates. Analysis of a larger set of isolates, particularly from food, animal and other parts of the environment, with the inclusion of both sporadic and outbreak-related isolates, as well as patients’ demographics related to human isolates over a larger time period, is recommended in order to obtain a more comprehensive picture of the molecular epidemiology of *S*. Enteritidis.

## Figures and Tables

**Table 1:** Number of *S.* Enteritidis isolates and respective sequence types.

**Types of Samples**	**Isolates (*n*)**	**ST1925 (*n*)**	**ST11 (*n*)**
**Human**	**246**	**184**	**62**
Human (intestinal)	171	135	36
Human (extra-intestinal)	75	49	26
**Food**	**27**	**8**	**19**
Chicken meat	23	6	17
Cooked or ready-to-eat food	2	1	1
Duck meat	2	1	1
**Farm and slaughter house environment**	**3**	**2**	**1**
Environmental swab (drag swab/farm)	2	2	0
Water (ice block/slaughter house)	1	0	1

**Table 2:** Antimicrobial resistance-related genes in *S*. Enteritidis isolates (*n* = 276).

		Human (*n* = 246)		Food (*n* = 27)		Animal (*n* = 3)		
		Intestinal (*n* = 171)	Extra-Intestinal (*n* = 75)	Chicken (*n* = 23)	Non-Chicken (*n* = 4)	Environmental Swab (*n* = 2)	Water (*n* = 1)	Total
Aminoglycosides	*Aac6-Iaa_AGly*	171	75	22	4	2	1	276
	*Aac3-Iva_AGly*	1	0	0	0	0	0	1
	*AadA_AGly*	1	0	0	0	0	0	1
	*Aph4-Ia_AGly*	1	0	0	0	0	0	1
Tetracycline	*tetA_tet*	108 *	33	5	3	2	1	153
Beta-lactam	*TEM-1D_bla*	12	16 *	7	1	0	0	36
	*CMY_bla*	2	0	0	0	1	0	3
	*CTX-M-1_bla*	1	1	0	0	0	0	2
Polymyxin	*mcr1_colistin*	1	0	0	0	0	0	1
Fluoroquinolone	*qnr-S_flq*	1	0	0	0	0	0	1
Folate pathway inhibitor	*SulII_sul*	0	0	0	0	0	1	1
	*SulIII_Sul*	1	0	0	0	0	0	1

* *p* < 0.05 by Chi-Square test.

## Data Availability

Not applicable.
